# Administration of non-national immunization program vaccines for children under six in a rural county, Henan Province: Did costs matter?

**DOI:** 10.1080/21645515.2025.2454744

**Published:** 2025-01-21

**Authors:** Miaomiao Yin, Yuan Cao, Xiaolin Xu, Hanzhi Peng, Yu Wang, Qian Long

**Affiliations:** a Global Health Research Center, Duke Kunshan University, Kunshan, Jiangsu, China; b School of Public Health, The Second Affiliated Hospital, Zhejiang University School of Medicine, Hangzhou, Zhejiang, China; c Infectious Disease Control Department, Song County Center for Disease Control and Prevention, Luoyang, Henan, China; d Global Health Research Center, Division of Social Sciences, Duke Kunshan University, Kunshan, Jiangsu, China; e Duke Global Health Institute, Duke University, Durham, NC, USA

**Keywords:** Non-national immunization program vaccines, left-behind children, rural area, administration, China

## Abstract

This study aimed to investigate caregivers’ administration of non-National Immunization Program (NIP) vaccines in rural China, and examine health system, individual, and social determinants. A cross-sectional survey (*n* = 1051) was conducted from July to October in 2022 in a rural county of Henan Province. Caregivers of children under six who came to township health centers for child vaccination were interviewed. Cross-tabulation and multivariate logistic regressions were used to examine the administration rate and associated factors. Qualitative interviews were conducted with healthcare professionals (*n* = 4) and caregivers (Focus Group Discussions, *n* = 4) to understand local policies, routine practices, and caregivers’ experience with the administration of non-NIP vaccines. A framework approach was used to analyze qualitative data. Quantitative and qualitative data were integrated during the interpretation of the results. The administration rate for non-NIP vaccines remained low in rural Henan, 26.7% of children had not received any non-NIP vaccines, 43.5% had received 1–2 types, and around 30% had received 3–5 types. There were no significant differences in the administration of non-NIP vaccines between left-behind and non-left-behind children after adjusting for characteristics of children, caregivers, and households. Qualitative findings reflected that the high cost of non-NIP vaccines was a primary factor influencing caregivers’ vaccination decision. Poor communication between physicians and caregivers was another significant factor, which was caused by low retention of healthcare workers, a shortage of professionals, and insufficient financial incentives for physicians. This study enriches the evidence on non-NIP vaccination among rural children, particularly for the vulnerable group of left-behind children.

## Introduction

Vaccines are viewed as one of the most cost-effective public health interventions to prevent deaths and improve lives, especially for children.^1^ In 1978, China established National Immunization Program (NIP), which has achieved over 90% coverage at the county level since 2012.^2^ Vaccines under the NIP are government-funded and mandatory. However, some important vaccines recommended by World Health Organization (WHO), such as Haemophilus influenzae type b (Hib), pneumococcal, rotavirus, and varicella vaccines are not included in China’s NIP. Unlike NIP vaccines, non-NIP vaccines are administered voluntarily and paid out-of-pocket.^3^ The uptake of non-NIP vaccines in China remains relatively low. A national survey conducted in 2019 reported that the first-dose coverage was highest for varicella vaccine (67.1%), followed by Hib vaccine (42.6%), enterovirus 71 (EV71) vaccine(39.5%) and rotavirus vaccine (20.3%), while pneumococcal conjugate vaccine (PCV) had the lowest coverage at 7.7%.^4^

Significant disparities in non-NIP vaccine coverage exist across socio-economic regions and between urban and rural areas in China. A meta-analysis of 97 studies in China found higher administration rates for pneumococcal vaccines in the more developed eastern and central regions compared to the less developed western region.^5^ Similarly, studies conducted in several eastern provinces reported higher administration rates for non-NIP vaccines among urban children compared to their rural or suburban counterparts.^6,7^

With rapid economic development and urbanization in China, many rural parents migrate to cities for work, leading to a large population of left-behind children.^8^ Previous studies have shown that left-behind children are more likely to get sick or develop chronic conditions, experience poor health-related quality of life in physical and psychological domains and reduce health services utilization.^9–11^ Studies conducted in central and western provinces found that left-behind children experienced lower timely administration and longer delays in receiving NIP vaccines compared to their non-left-behind counterparts.^12,13^ However, there is a lack of research on the administration of non-NIP vaccines among left-behind children in China. These findings highlight the urgent need to address the immunization status of this vulnerable population.

Globally, various individual and family-level factors influencing vaccine uptake have been identified. A meta-analysis of 36 studies from Asia, the Americas, Europe, and Oceania found that older parental age, lower education levels, and lower household income negatively impacted seasonal influenza vaccine uptake, while support from relatives and recommendations from healthcare professionals had positive effects.^14^ Another systematic review of 30 studies from eight countries indicated that caregivers’ knowledge, positive attitudes, and healthcare worker recommendations positively influenced children’s influenza vaccination, while concerns about safety, side effects, and poor access to vaccination services were barriers.^15^ In China, a scoping review and other studies identified that high vaccine costs, low disease awareness, safety concerns, and insufficient caregivers’ knowledge of vaccination were key obstacles to the administration of non-NIP vaccines.^16–18^

This study compared the administration of several non-NIP vaccines among left-behind and non-left-behind children in rural areas, as well as explored the health system, individual, and social determinants influencing administration of non-NIP vaccines. Based on previous findings, we hypothesized that left-behind children, as a vulnerable group, are less likely to receive non-NIP vaccines than their counterparts.

## Method

### Study design and setting

This study was carried out as a part of the project studying “Immunization coverage, knowledge, satisfaction, and associated factors of non‑National Immunization Program vaccines among migrant and left‑behind families in China: evidence from Zhejiang and Henan provinces.” This design is primarily a mixed-methods approach, with the integration of qualitative and quantitative data occurring mainly during the interpretation of the results.

The study was conducted in a rural county of a central province, Henan. The study county hosted 541,000 residents in 2022 with a total of 41,242 children aged 0–6 years old. In 2022, the county’s Gross Domestic Product (GDP) per capita was $6338.70 USD, which was below both the provincial average ($9233.58 USD) and the national average ($12741.11 USD). The annual per capita disposable income of local rural residents was $2,419.08 USD, lower than both the provincial average of $2,777.09 USD and the national average of $2,993.27 USD. This study examined the administration of five non-NIP vaccines for children under six: varicella vaccine, Hib vaccine, EV71 vaccine, 13-valent pneumococcal conjugate vaccine (PCV13), and rotavirus vaccine. These vaccines were selected due to their relevance to the current public health concerns and their availability and accessibility at the study site.

### Quantitative study

#### Data collection

A cross-sectional survey was conducted from July to October in 2022. Out of the sixteen townships in the study county, five were selected based on the criteria that included a large number of children under six, a significant proportion of left-behind children (defined as one or both parents migrating into cities for work, leaving their children in the rural communities with other caregivers like grandparents for over six months), and the willingness of local township health centers to cooperate. The study population was caregivers of children under six including parents and/or grandparents, who were invited to participate in the survey during their visit to the township health center for child vaccination. Caregivers were excluded if their children were not able to be vaccinated on the day of the survey due to contraindications, or other health-related issues or if they were not involved in vaccination decision-making.

The questionnaire included questions on caregivers’ and children’s demographic information and socioeconomic status, caregivers’ health status, sources of vaccination-related information, caregivers’ acceptance and administration of both NIP and non-NIP vaccines, and their satisfaction with children’s vaccination. Before the formal survey, a pretest was conducted with 10 participants, including five grandmothers of left-behind children and five mothers of non-left-behind children to refine and finalize the questionnaire. Physicians and students who received standardized training conducted face-to-face interviews with caregivers using an online platform. The team member (CY) reviewed the submitted questionnaires every week, focusing on completeness, logical consistency, and data standardization, and returned the identified errors to the investigators for correction by reaching out to caregivers again if possible. Prior to the survey, all participants were provided with informed consent materials through the online platform. A total of 1,106 caregivers were invited to participate in the survey, 55 participants did not meet the inclusion criteria and were excluded from this analysis.

#### Data analysis

The outcome measure was the administration of non-NIP vaccines, which was categorized into three groups: did not receive any non-NIP vaccines, received 1–2 types of non-NIP vaccines, and received 3–5 types of non-NIP vaccines. The administration of non-NIP vaccines was verified by asking caregivers if the child had received at least one dose of these vaccines. Explanatory variables were 1) children’s characteristics including their age, sex, and parental migration status; 2) caregivers’ characteristics including their age, sex, and education level; 3) family background including the number of children (1, 2 or ≥ 3) and annual household income. The annual household income was divided into three groups equally, representing low, medium, and high levels of income.

Cross-tabulation was used to compare the characteristics of children, caregivers, and families, as well as the vaccination rate of non-NIP vaccines by parental migration status. The chi-square test and student’s t-test were employed to test the statistical difference between left-behind and non-left-behind children. Based on the results of descriptive analysis, two multivariable logistic regression analyses were conducted to identify association between parental migration status (left-behind vs. non-left-behind children) with administration of non-NIP vaccines after adjusting for other children, caregivers, and family characteristics. The first analysis categorized the outcome as receiving no non-NIP vaccines versus receiving at least one. The second analysis categorized the outcome as receiving 1–2 types versus 3–5 types of non-NIP vaccines. Odds ratios (ORs) and 95% confidence intervals (CIs) were calculated to examine the associations between child and caregiver characteristics and non-NIP vaccination. All analyses were performed using Stata (Version MP 17.0).

### Qualitative study

#### Data collection

We selected two townships: one above and one below the county average per capita disposable income to conduct the qualitative interviews. Semi-structured individual in-depth interviews were carried out with the head of the immunization department at the county Center for Disease Control and Prevention (CDC) and three healthcare providers at the township health center who are in charge of or participate in immunization services. These interviews aimed to understand local policies and practices for non-NIP vaccines, including advocacy efforts, vaccine procurement, and physician training, as well as healthcare professionals’ perceptions of factors affecting the caregivers’ decision-making regarding the administration of non-NIP vaccines. Additionally, four focus group discussions (FGD) were held with caregivers of children under six, comprising three FGDs with caregivers of left-behind children, and one FGD with caregivers of non-left-behind children, each group consisting of six to seven interviewees. To ensure diversity, we included caregivers with different roles, such as grandparents and parents, as well as those with one child or two or more children. These were to understand caregivers’ attitudes, willingness, and experiences related to non-NIP vaccinations.

The qualitative interviewer was a health system researcher with extensive experience in conducting qualitative interviews. The two other researchers served as note-taker and observer, both trained in qualitative methods and ethical considerations related to qualitative studies. The interviews with healthcare professionals were conducted in their offices. Caregivers were invited to the local township health center by a physician, where FGDs were conducted in a meeting room. With the consent of all participants, the interviews were recorded and lasted 40–50 minutes.

#### Data analysis

All interviews were transcribed into Chinese. The framework approach was used to analyze qualitative data. An analytical framework, informed by the topic guide and the categories that emerged from the transcripts, was developed and applied to the data to identify themes. All data were coded, sorted, and classified using NVivo v.12 (Lumivero, Denver, CO, USA). A charting table was used to identify common or divergent perceptions within and across different stakeholders. The interviewer summarized the key qualitative findings, which were then collectively discussed with other team members and experts in the study topic to ensure a comprehensive understanding and validation of the results.

## Results

### Participants of quantitative and qualitative study

Table 1 presents the demographic and socioeconomic characteristics of participants in the quantitative survey. Among the 1051 participants, 392 were left-behind children and 659 were non-left-behind children. The average age of children was 2.72 years. Left-behind children were moderately older than non-left-behind children (*p* < .01). The proportion of left-behind female children (52.3%) was slightly higher than that of their non-left-behind counterparts (49.6%), but the difference was not statistically significant. A vast majority of caregivers were female. Over half of the left-behind children (58.4%) were cared for by their grandparents, compared to 4.4% of their counterparts. 45.4% of caregivers of left-behind children received primary or lower education, while this proportion was only 8.8% among the caregivers of non-left-behind children. Regarding family size and household income, approximately one-third of the left-behind children had two or more siblings and came from high-income households, in contrast to their peers, of whom only 25.0% had multiple siblings and 24.9% came from similarly affluent families.

**Table 1.** Demographic and socioeconomic characteristics of family by parental migration status in rural Henan, 2022.

	Total		Left-behind		Non-left-behind		χ2/T	P
	(*n*=1051)		(*n*=392)		(*n*=659)			
	No.	%	No.	%	No.	%		
**Children’s characteristics**								
**Sex**							0.704	0.402
Male	519	49.38	187	47.7	332	50.38		
Female	532	50.62	205	52.3	327	49.62		
**Age**							−5.641	<0.001
Mean (SD)	2.72 (1.69)		3.09 (1.54)		2.5 (1.74)			
**Caregiver’s characteristics**								
**Sex**							4.890	0.027
Male	182	17.32	81	20.66	101	15.33		
Female	869	82.68	311	79.34	558	84.67		
**Age**^**a**^							333.232	<0.001
≤30	255	24.26	51	13.01	204	30.96		
30–50	565	53.76	138	35.2	427	64.8		
>50	228	21.69	203	51.79	25	3.79		
**Relationship with children**							387.217	<0.001
Parents	793	75.45	163	41.58	630	95.6		
Grandparents	258	24.55	229	58.42	29	4.4		
**Education**							191.226	<0.001
Primary and lower	236	22.45	178	45.41	58	8.8		
Middle school	563	53.57	157	40.05	406	61.61		
High school and higher	252	23.98	57	14.54	195	29.59		
**Family size and household income**								
**Number of children**^**b**^							7.784	0.020
1	436	41.48	164	41.84	272	41.27		
2	322	30.64	103	26.28	219	33.23		
≥3	289	27.5	124	31.63	165	25.04		
**Annual household income (USD)**^**c**^							6.537	0.038
Low (≤$4459.83 USD)	379	36.48	140	35.81	239	36.88		
Middle ($4459.83–7433.06 USD)	375	36.09	127	32.48	248	38.27		
High (>$7433.06 USD)	285	27.43	124	31.71	161	24.85		

^a^Data were missing for three families; ^b^Data were missing for four families; ^c^Data were missing for twelve families. 2022 average currency exchange rate: $1 USD =6.7261 RMB.

Four healthcare professionals participated in in-depth interviews. Two physicians had been providing immunization services at township health centers for around one year. The heads of the immunization departments at the township health center and the county CDC had over 10 years of experience in the field. Regarding the FGDs, one focused on caregivers of non-left-behind children, including seven mothers aged 26 to 35 years, with four mothers having three children, one mother having two children, and two mothers having one child. The other three FGDs focused on left-behind children, comprising a total of seventeen grandparents and three mothers, the majority of these families had at least two children.

### Regulation of non-NIP vaccine delivery in Henan

In 2021, the provincial health commission of Henan province issued a practical guideline for non-NIP vaccines, aligned with the national guideline for non-NIP vaccines (2020 version) and the immunization program standards. The guideline prioritizes completing NIP vaccinations and emphasizes the voluntary choice of non-NIP vaccine administration. Healthcare providers are required to provide necessary information and record non-NIP vaccinations in the provincial and national immunization program information systems.

The qualitative interviews with healthcare professionals involved in local immunization services indicated that the procurement of non-NIP vaccines depended on caregivers’ “willingness” to vaccinate their children. The county CDC was in charge of purchasing non-NIP vaccines through a provincial vaccine supply platform according to the type and estimated quantity of non-NIP vaccines submitted by local vaccination sites. An official electronic application (APP), “Xiao Dou Miao,” was developed to streamline vaccination services. This APP displays information related to NIP and non-NIP vaccines and has a reminder function according to the immunization schedule. The head of the immunization department at the county CDC said that parents of newborns were recommended to install this APP, and the majority complied.

Table 2 presents doses, schedules, and prices per dose of five non-NIP vaccines in the study county. The price per dose of PCV13 is the highest ($71.36 USD), followed by rotavirus vaccine ($44.90 USD). Hib, varicella, and EV71 vaccines ranged from $18.44 USD to $28.25 USD. The total cost for a child receiving the full series of PCV13 (four doses starting at two months of age), rotavirus, Hib, varicella, and EV71 vaccines amounted to $597.37 USD, which is equivalent to 24.69% of the annual per capita disposable income of local rural residents.

**Table 2.** Recommended schedule and price of five non-nip vaccines in Henan, 2022.

Vaccine		Doses in Primary Series	Schedule				Recommended Vaccination Age	Cost/Dose (USD)	Total Cost (USD)
			1^st^ dose	2^nd^ dose	3^rd^ dose	4^th^ dose			
Hib vaccine		4	3 months	4 months	5 months	18 months	2 months-5 years	18.44	73.74
Varicella vaccine		2	1 year	4 years			>1 year	23.49	46.98
EV71 vaccine		2	6 months	7 months			6 months-3 years	28.25	56.50
Rotavirus vaccine		3	6–10 weeks	10–22 weeks	<32 weeks		6–32 weeks	44.90	134.70
PCV13 vaccine	Option 1	4	2 months	3 months	4 months	12 months	6 weeks-12 months	71.36	285.46
	Option 2	2	≥12 months	≥14 months			12–23 months		143.02
	Option 3	1	2 years (min)				2–5 years		71.66

Hib, Haemophilus influenza type b vaccine; EV71, enterovirus type 71 vaccine; PCV13, 13-valent pneumococcal conjugate vaccine.

2022 average currency exchange rate: $1 USD = 6.7261 RMB

### Information source of vaccination-related knowledge

The quantitative survey asked three questions about the vaccination schedule. Almost all caregivers knew that vaccination should adhere to the recommended schedule but could be deferred or contraindicated according to the child’s health status. While one-fifth of caregivers of left-behind children did not know multiple doses are required for vaccines to achieve full effectiveness. This proportion was lower among caregivers of non-left-behind children (8.4%) (Supplementary Table S1).

According to the quantitative survey, the primary vaccination-related information sources for caregivers were healthcare professionals (90.9%), followed by mobile applications and the internet (71.7%), and local offline health education activities (53.6%). Caregivers of left-behind children were more likely to acquire information from mobile applications and the internet, while less likely to participate in local health education activities compared to their counterparts (Supplementary Table S2).

The qualitative results were consistent with the quantitative findings. Most caregivers and all healthcare professionals indicated that physicians explained to vaccination-related information caregivers, such as vaccine-preventable diseases (both NIP and non-NIP vaccines), potential side effects, management of adverse reactions, and immunization schedule during vaccination visits. In addition, this information was also disseminated through the offline “Mom classes” organized by the vaccination sites and the official APP “Xiao Dou Miao.” Some grandparents of left-behind children mentioned that parents used the “Xiao Dou Miao” App to study vaccines. One mom of a non-left-behind child said she primarily acquired vaccine-related knowledge through the official APP. She also sourced relevant information from other social media, though with skepticism regarding the veracity of the information. Almost all healthcare professionals indicated that they informed caregivers about choices of non-NIP vaccines but would not “recommend” them primarily due to the requisite out-of-pocket expenses that may foster caregiver mistrust and complaints.

*“(the vaccine-related knowledge was disseminated) through Mom classes and village doctors. Mom classes are held once a month. We introduce each type of (non-NIP) vaccine to caregivers according to their child’s age and let them choose. We keep informing them if their child is still within the eligible age (for vaccination). But if their child missed the recommended schedule, we would not talk anymore. (Physician at the township health center in DT township, in-depth interview)*

*I have attended a Mom class before. There is someone who specifically talks about prevention knowledge, and the benefits of NIP and non-NIP vaccines, after the talk, they give you a book (vaccination record). (A grandmother of a left-behind child in DZ township, FGD)*

*(Their parents) make a phone call to talk about it. Young people all have the Xiaodoumiao app on their phones, so they know which vaccines need to be given. It shows both the NIP vaccines and non-NIP vaccines. (A grandmother of a left-behind child in DZ township, FGD)*

*Being a mother for the first time, I’m quite cautious. I searched for information through pregnancy apps, Xiaodoumiao app (mainly), and TikTok. Mostly, I rely on Xiaodoumiao app; TikTok is just for reference, I don’t trust it completely. (A mother of a non-left-behind child in DT township, FGD)*

### Administration of non-NIP vaccines and factors associated with administration of non-NIP vaccines

The quantitative survey found that 29.6% of non-left-behind children had not received any non-NIP vaccines, compared to 21.9% of left-behind children (Table 3). Almost half of left-behind children and 39.8% of non-left-behind children had received one or two types of non-NIP vaccines. Around 30% of children had received more than two types of non-NIP vaccines with no significant differences between left-behind and non-left-behind children.

**Table 3.** The administration rates of non-nip vaccines among children under six in rural Henan, 2022.

	Total (n)		left-behind (n_a_)		non-left-behind (n_b_)		χ2	P
	N	%	N	%	N	%		
Not vaccinated	281	26.74	86	21.94	195	29.59	7.345	0.007
Vaccinated 1–2 types	457	43.48	195	49.74	262	39.76	9.977	0.002
Vaccinated 3–5 types	313	29.78	111	28.32	202	30.65	0.642	0.423
**The administration rate of each type of non-NIP vaccine**								
Hib (*n*=1038, n_a_=392, n_b_ =646)	617	59.44	241	61.48	376	58.2	1.086	0.297
Varicella (*n*=810, n_a_=188, n_b_ =465)	450	55.56	188	54.49	262	56.34	0.275	0.600
EV71 (*n*=951, n_a_ =380, n_b_=571)	380	39.96	155	40.79	225	39.4	0.182	0.669
Rotavirus (*n*=1051, n_a_ =392, n_b_ =659)	263	25.02	77	19.64	186	28.22	9.648	0.002
PCV13 (*n*=1051, n_a_ =392, n_b_ =659)	122	11.61	35	8.93	87	13.2	4.374	0.036

Hib, Haemophilus influenza type b vaccine; EV71, enterovirus type 71 vaccine; PCV13, 13-valent pneumococcal conjugate vaccine.

Among the five non-NIP vaccines, the highest administration rates were observed for Hib vaccine (59.44%), followed by varicella vaccine (55.56%) and EV71 vaccine (39.96%). There was no significant difference in the administration rate among left-behind and non-left-behind children. Lower rates were observed for rotavirus and PCV13 vaccines, which were also more expensive. Rotavirus vaccine was administered to 19.64% of left-behind children and 28.22% of non-left-behind children (p < .05). The lowest administration rate was for PCV13, with 8.93% of left behind children and 13.20% of non-left-behind children being vaccinated (p < .05). (Table 3)

Table 4 summarizes the results from the multivariate logistic regulation model. After adjusting for all variables, left-behind children were more likely to receive at least one dose of non-NIP vaccines than non-left-behind children, although the difference was not statistically significant (*OR* = 1.21 95% *CI*: 0.84–1.75). The likelihood of receiving at least one dose of non-NIP vaccines was negatively associated with younger age (OR = 1.41 95% CI: 1.28–1.55) and having more than one sibling (OR = 0.67 95% CI:0.48–0.94). Children from high-income families were less likely to receive non-NIP vaccines compared to those from low-income families (OR = 0.58 95% CI: 0.40–0.83). Additionally, older children were more likely to receive multiple non-NIP vaccines (OR = 1.25 95%CI:1.13–1.37). Female children were less likely to receive more than two types of non-NIP vaccines compared to male children (OR = 0.66 95% CI: 0.49–0.89).

**Table 4.** Factors associated with non-nip vaccine administration among children under six in rural Henan, 2022.

	Model 1 (total, *n*=1033)					Model 2 (vaccinated, *n*=758)			
	OR	95%CI		P		OR	95%CI		P
**Children’s characteristics**									
**Category**									
Non-left-behind	Reference					Reference			
Left-behind	1.21	0.84	1.75	0.303		0.74	0.51	1.09	0.126
**Sex**									
Male	Reference					Reference			
Female	1.02	0.76	1.36	0.912		0.66	0.49	0.89	0.007
**Age**	1.41	1.28	1.55	<0.001		1.25	1.13	1.37	<0.001
**Caregiver’s characteristics**									
**Sex**									
Male	Reference					Reference			
Female	1.05	0.71	1.56	0.804		0.91	0.61	1.37	0.655
**Age**									
≤30	Reference					Reference			
30–50	0.80	0.56	1.15	0.232		0.80	0.54	1.18	0.255
≥50	1.05	0.58	1.91	0.866		0.59	0.32	1.08	0.089
**Education**									
Primary and lower	Reference					Reference			
Middle school	1.16	0.73	1.84	0.543		0.72	0.45	1.15	0.167
High school and higher	1.10	0.65	1.88	0.721		0.67	0.38	1.17	0.160
**Family background**									
**Number of Children**									
1	Reference					Reference			
2	0.67	0.48	0.94	0.020		1.01	0.70	1.46	0.960
3	0.98	0.67	1.43	0.921		0.82	0.57	1.19	0.300
**Annually Household Income**									
Low	Reference					Reference			
Middle	1.10	0.77	1.56	0.607		1.40	0.99	1.99	0.057
High	0.58	0.40	0.83	0.003		0.96	0.64	1.43	0.832

Model 1: whether the children has been vaccinated non-NIP vaccines. Model 2: Children vaccinated with one to two non-NIP vaccines or more than three non-NIP vaccines.

Consistent with quantitative findings, almost all healthcare professionals indicated that the most widely administered non-NIP vaccines were varicella and EV71 vaccines. The administration of rotavirus and PCV13 vaccines was generally low, primarily due to high expenses and financial pressure faced by caregivers, as perceived by almost all healthcare professionals. In addition, the head of immunization department at the county CDC also pointed out that poor communication between physicians and caregivers hindered demands for non-NIP vaccines, which was attributed to low retention and insufficient qualified health workforce at the vaccination sites. Moreover, there was a lack of financial incentive for physicians to promote non-NIP vaccines.

*We administer over 100 doses of rotavirus vaccine and 300–400 doses of PCV13 a month, which is far less compared to EV71 and varicella vaccines, for which we use over 1000 doses each month. … The main reasons are the cost and poor promotion. The lower usage of rotavirus vaccine might be due to its price, and because it hasn’t been in use for a long time, resulting in less awareness and understanding of it. … In rural township health centers, some vaccination doctors are replaced twice a year, so they lack the professional competence. My training even can’t keep up with their turnover. Their low professional ability means they can’t explain the benefits of some vaccines, so the public doesn’t accept and get vaccinated…. For non-immunization program vaccines, there is no promotion, and the 14-yuan service fee does not actually benefit individuals but goes directly to the operating funds of township health centers. (The head of the Immunization Department at the Center for Disease Control and Prevention in the studied county, in-depth interview)*

*Awareness is quite high, but since these are self-paid vaccines, sometimes when we ask parents if they want to vaccinate their children, some refuse as soon as they hear it costs money. However, most will listen and say that they’ll come back when they have money. Due to the pandemic over the past three years, many people have been stuck at home and unable to work outside, leading to high household expenses, which make it very difficult for them to live. (Physician at the township health center in DT township, in-depth interview)*

*Price is one factor, and awareness is another. Some parents specifically request self-paid vaccines, and these parents usually have jobs and are not just staying at home. I think the main issue is still price. It’s not that the parents think the vaccine is unimportant, but they are unwilling to pay for it. If it were free, they would definitely get it. If the price were lower, the parents might also accept it, the price below 200 yuan would be more acceptable. (Physician at the township health center in DZ township, in-depth interview)*

Most caregivers of both non-left-behind and left-behind children said financial affordability and perceived severity of diseases were major factors impacting their decision-making. Most mothers with two or more children also indicated the growth in the number of children in a family often resulted in increased financial pressure, which in turn impacted their choice of vaccines when out-of-pocket payment was required.

*If I have the money, I will get my child vaccinated. If I don’t have the money, I can’t. It’s mainly about the money.” (A mother of a left-behind child in DT township, FGD)*

*Depending on the severity of the disease, if it is particularly serious, it must be prevented. If it’s not too expensive, we’ll get the vaccine. Even though the 13-valent pneumonia vaccine is costly, we’ve still gotten it. (A mother of a left-behind child in DZ township, FGD2)*

*But if it’s too expensive, we can’t accept. If there is a free vaccine, we will get the free one, even if it required more trips. If our economic situation improves, we’ll get the self-paid vaccine, if not, we don’t. Even the shot that cost 300 yuan is a stretch, since our economic situation is limited. (A mother of a left-behind child in DZ township, FGD1)*

*In recent years, the economy is particularly bad, and prices are very high…… For self-paid vaccines, we have to think very carefully. (A mother of a non-left-behind child in DT township, one child, FGD1)*

*We definitely want to get vaccinated, but every time we come, we feel a bit expensive, especially with multiple children. If it were free, we would certainly get vaccinated. For our children’s health, we would get them. (A mother of a non-left-behind child in DT township, FGD)*

*There are many children at home, it’s a bit expensive, so we can’t afford it. (A mother of a non-left-behind child in DT township, multiple children, FGD)*

## Discussion

This study investigated the administration of non-NIP vaccines among left-behind and non-left-behind children, as well as health system and social determinants of the non-NIP vaccines administration in rural Henan Province. Overall, the administration rate of non-NIP vaccines was low, with no significant differences observed between the two groups after adjusting for child, caregiver, and household characteristics. This finding was consistent across both quantitative and qualitative results. Qualitative findings indicated that the procurement and allocation of non-NIP vaccines were demand-driven, while high vaccine costs and poor communication between physicians and caregivers contributed to the low administration rates.

Quantitative results showed that the administration rates for Hib, varicella, and EV 71 vaccines ranged from 40% to 60%, comparable to previous studies.^4,19^ Qualitative results revealed that EV71 and varicella vaccines were particularly popular due to caregivers’ high perceived susceptibility to hand, foot, and mouth disease and varicella, with similar results also found in other studies.^20,21^ In this study, only 28% of eligible non-left-behind children and 20% of left-behind children received rotavirus vaccine, much lower than the administration rate in municipality of Shanghai at 46.2%.^22^ About 12% of children received PCV13, which was lower than the 20% first-dose coverage reported in other rural areas across nine provinces in 2021.^23^ Overall, the administration rates for these five vaccines in China, especially in rural areas, were far below than the global average and some other low- and middle-income countries (LMICs), where coverage generally exceeds 80%.^24^ The low rates reflect the combined impact of supply-side constraints and demand-side challenges.

Quantitative and qualitative results indicated that physicians were the most primary and trusted source of vaccine-related information for caregivers. Qualitative interviews reflected that some physicians at rural township health centers engaged in immunization services were temporary workers. Low salaries and limited career development contributed to a low retention rate and insufficient qualified health workforce in grassroots level, which is consistent with findings in other studies conducted in rural China.^25^ In addition, because non-NIP vaccination is voluntary and requires out-of-pocket payment, physicians often hesitate to recommend them to avoid caregivers’ misunderstandings that they are promoting vaccine sales. These issues negatively impact effective communication, leading to caregivers’ insufficient understanding and hesitancy in non-NIP vaccination. Moreover, other studies have reported that the heavy workload associated with providing essential public health services, along with financial disincentives for physicians administering immunizations, also impedes the promotion of non-NIP vaccines.^25,26^

Qualitative findings highlighted the cost of vaccines as the primary concern for caregivers, consistent with previous studies.^27,28^ In 2022, rural areas’ per capita disposable income was only 40.85% of urban areas’ income, and the cost of a full dose of five selected non-NIP vaccines would consume nearly a quarter of a rural household’s annual income, creating a significant financial burden. Logistic regression results showed that families with more than one child were less likely to administer non-NIP vaccines, possibly due to financial pressure. In addition, older children tended to receive more non-NIP vaccines, potentially because caregivers delay non-NIP vaccines to avoid overloading younger children with vaccinations. Female children were less likely to receive more non-NIP vaccines than male children, although this was inconsistent with qualitative findings. Further studies to investigate the impact of child sex, sibling structure, and other social determinants on non-NIP vaccination in rural China are needed, aiming to improve equitable access to vaccination services.

Our study found that the “Xiaodoumiao” app is a potentially effective tool for disseminating and managing immunization information in Henan Province. To improve caregivers’ knowledge and acceptance, this type of new approach should be developed and assessed for scale-up, especially in rural or less developed areas. Given the affordability of the rural population, a multi-source financing mechanism like co-payment of public funds and personal contributions should be explored. Moreover, implementing a strategic purchasing mechanism for non-NIP vaccines, like centralized drug procurement, could reduce costs and improve equity in terms of access and affordability of vaccines. Strengthening the capacity of immunization service delivery through targeted training and introducing financial and non-financial incentives is also critical for motivating the health workforce to enhance effective communication with caregivers, build trust, and promote vaccine uptake.

This study enriches the evidence on non-NIP vaccination among rural children, particularly for the vulnerable group of left-behind children. However, there are some limitations in our study. First, the proportion of left-behind children in the sample was relatively small and unbalanced compared to non-left-behind children, potentially introducing selection bias. Therefore, the generalizability of the findings, especially vaccination among left-behind children should be made with caution. Second, household economic status was assessed based solely on total annual income without considering per capita income, which may have confounded the association between income level and vaccine uptake. Third, qualitative interview participants were recruited by local physicians, potentially leading to a selection bias favoring those with greater trust in healthcare providers; they may have a better understanding and good adherence to child vaccination schedules.

## Conclusion

The overall administration rate for non-NIP vaccines remained low in rural Henan Province without significant differences in the administration of non-NIP vaccines between left-behind and non-left-behind children. The cost of the non-NIP vaccines was a major determinant of the administration, and poor communication between physicians and caregivers, caused by low retention of healthcare workers, a shortage of professionals, and insufficient financial incentives for physicians, was another significant factor impacting caregivers’ demands.

## Supplementary Material

Additional file.docx

## Data Availability

The dataset and analysis script are available upon reasonable request to the corresponding author.

## Abbreviations

NIP: National Immunization Program
WHO: World Health Organization.
GDP: Gross Domestic Product
Hib: Hemophilus influenza type b vaccine
EV71: Enterovirus type 71 vaccine
PCV13: 13-valent pneumonia vaccine
OR: Odds ratio
CI: Confidence interval
CDC: Center for Disease Control and Prevention
FGD: Focus group discussion
APP: Application
LMIC: low- and middle-income country

## References

[1] Turner HC, Thwaites GE, Clapham HE. Vaccine-preventable diseases in lower-middle-income countries. Lancet Infect Dis. 2018;18(9):937–9. doi:10.1016/S1473-3099(18)30478-X. 30152349

[2] Pan J, Wang Y, Cao L, Wang Y, Zhao Q, Tang S, Gong W, Guo L, Liu Z, Wen Z, et al. Impact of immunization programs on 11 childhood vaccine-preventable diseases in China: 1950–2018. Innovation. 2021;2(2):100113. doi:10.1016/j.xinn.2021.100113. 34557762 PMC8454656

[3] Chen S, Yao L, Wang W, Tang S. Developing an effective and sustainable national immunisation programme in China: issues and challenges. Lancet Public Health. 2022;7(12):e1064–e1072. doi:10.1016/s2468-2667(22)00171-2. 36252582 PMC9712122

[4] Zhang HJ, Lai XZ, Mak J, Sriudomporn S, Zhang HN, Fang H, Patenaude B. Coverage and equity of childhood vaccines in China. Jama Network Open. 2022;5(12):e2246005. doi:10.1001/jamanetworkopen.2022.46005. 36484985 PMC9856225

[5] Shao W, Chen X, Zheng C, Wang G, Zhang B, Zhang W. Pneumococcal vaccination coverage and willingness in Mainland China. Trop Med Int Health. 2022;27(10):864–872. doi:10.1111/tmi.13809. 35942809

[6] Zhang XY, Syeda ZI, Jing ZY, Xu QQ, Sun L, Xu LZ, Zhou C. Rural-urban disparity in category II vaccination among children under five years of age: evidence from a survey in Shandong, China. Int J Equity Health. 2018;17(1):17. doi:10.1186/s12939-018-0802-4. 29929503 PMC6013881

[7] Liu Y, Xu Y, Wang J, Che X, Gu W, Du J, Zhang X, Zhang X, Jiang W, Chen J, et al. Vaccination pattern of the 23-valent pneumococcal polysaccharide vaccine (PPV23) in Hangzhou, China: a coverage and adverse events following immunization of different age groups. Human Vaccines & Immunotherapeutics. 2021;17(1):157–161. doi:10.1080/21645515.2020.1765620. 32530728 PMC7872049

[8] Lyu L, Mei Z, Yan F, Wang X, Duan C. The status of rural children left-behind in China: 2010–2020. China Popul Develop Stud. 2024;8(2):97–111. doi:10.1007/s42379-024-00159-2.

[9] Li Q, Liu G, Zang W. The health of left-behind children in rural China. China Eco Rev. 2015;36:367–376. doi:10.1016/j.chieco.2015.04.004.

[10] Huang Y, Zhong X-N, Li Q-Y, Xu D, Zhang X-L, Feng C, Yang G-X, Bo Y-Y, Deng B. Health-related quality of life of the rural-China left-behind children or adolescents and influential factors: a cross-sectional study. Health Qual Life Outcomes. 2015;13(1):1–10. doi:10.1186/s12955-015-0220-x. 25888732 PMC4349722

[11] Ouyang Y, Zou J, Ji M, Zhang Y, Yuan T, Yang L, Lin Q. Study on the status of health service utilization among 3–5 years old left-behind children in poor rural areas of Hunan Province, China: a cross-sectional survey. Int J Environ Res Public Health. 2019;16(1):125. doi:10.3390/ijerph16010125. 30621227 PMC6339067

[12] Ni ZL, Tan XD, Shao HY, Wang Y. Immunisation status and determinants of left-behind children aged 12-72 months in central China. Epidemiol Infect. 2017;145(9):1763–1772. doi:10.1017/s0950268817000589. 28357974 PMC9203325

[13] Tang X, Geater A, McNeil E, Zhou H, Deng Q, Dong A, Li Q. Parental migration and children’s timely measles vaccination in rural China: a cross-sectional study. Trop Med Int Health. 2016;21(7):886–894. doi:10.1111/tmi.12719. 27137758

[14] Chan PS, Fang Y, Kawuki J, Chen S, Liang X, Mo PK, Wang Z. Parental acceptance, parental hesitancy, and uptake of seasonal influenza vaccination among children aged 6–59 months: a systematic review and meta-analysis. Vaccines (Basel). 2023;11(8):1360. doi:10.3390/vaccines11081360. 37631928 PMC10459009

[15] Han KY, Hou ZY, Tu SY, Liu MY, Chantler T, Larson H. Factors influencing childhood influenza vaccination: a systematic review. Vaccines. 2024;12(3):233. doi:10.3390/vaccines12030233. 38543867 PMC10975121

[16] Jiang M, Chen S, Yan X, Ying X, Tang S. The coverage and challenges of increasing uptake of Non-National immunization program vaccines in China: a scoping review. Infect Dis Poverty. 2023;12(1):114. doi:10.1186/s40249-023-01150-8. 38062480 PMC10704715

[17] Yang Y, Wang Y, Yang D, Dong S, Yang Y, Zhu X, Chen Y, Zhou Y, Jiang Q. Factors associated with uptake of Haemophilus influenzae type b vaccination in Shanghai, China. BMC Pediatrics. 2019;19(1):8. doi:10.1186/s12887-018-1374-6. 30616637 PMC6323772

[18] Qu S, Yang M, He W, Xie H, Zhou M, Campy KS, Tao X. Determinants of parental self-reported uptake of influenza vaccination in preschool children during the COVID-19 pandemic. Human Vaccines & Immunotherapeutics. 2023;19(3):2268392. doi:10.1080/21645515.2023.2268392. 37964617 PMC10653755

[19] Yang Y, Yang Y, Scherpbier RW, Zhu X, Chen Y, Zhou Y, Jiang Q. Coverage of Haemophilus influenzae type b conjugate vaccine for children in Mainland China: systematic review and meta-analysis. Pediatr Infect Dis J. 2019;38(3):248–252. doi:10.1097/inf.0000000000002132. 29957731

[20] Wang YY, Meng FY, Li JX, Li GF, Hu JL, Cao JQ, Yu Q, Liang Q, Zhu F. Willingness of parents to vaccinate their 6–60-month-old children with EV71 vaccines: a cross-sectional study in rural areas of northern Jiangsu Province. Human Vaccines & Immunotherapeutics. 2020;16(7):1579–1585. doi:10.1080/21645515.2020.1737465. 32209003 PMC7482733

[21] Li TG, Wang H, Lu Y, Li Q, Chen C, Wang DH, Li M, Li Y, Lu J, Chen Z, et al. Willingness and influential factors of parents to vaccinate their children with novel inactivated enterovirus 71 vaccines in Guangzhou, China. Vaccine. 2018;36(26):3772–3778. doi:10.1016/j.vaccine.2018.05.054. 29776754

[22] Maimaiti H, Lu J, Guo X, Zhou L, Hu LJ, Lu YH. Vaccine uptake to prevent meningitis and Encephalitis in Shanghai, China. Vaccines. 2022;10(12):2054. doi:10.3390/vaccines10122054. 36560463 PMC9787460

[23] Liu L, Zhang Z, Zhang X, Xu C, Song Y, Li L, Ye J, Wang Z, Liang H, Zhang W, et al. Coverage of 13-valent pneumococcal conjugate vaccine among children 0–15 months of age — 9 provinces, China, 2019–2021. China CDC Wkly. 2023;5(17):379–384. doi:10.46234/ccdcw2023.072. 37197448 PMC10184383

[24] World Health Organization. WHO Immunization Data portal. 2022 [cited 2024 June 27th]; https://immunizationdata.who.int/.

[25] Gong D, Jiang Q, Chantler T, Sun FY, Zou J, Cheng J, Chen Y, Li C, Sun M, Howard N. Health system barriers and facilitators to delivering additional vaccines through the national immunisation programme in China: a qualitative study of provider and service-user perspectives. Vaccines. 2021;9(5):476. doi:10.3390/vaccines9050476. 34066844 PMC8151436

[26] Lyu Y, Lai X, Ma Y, Fang H. Factors associated with recommendation behaviors of four Non-National immunization program vaccines: a cross-sectional survey among public health workers in China. Inf Dis Poverty. 2023;12(1):58–68. doi:10.1186/s40249-023-01142-8. PMC10559509 37805654

[27] Wu L, Huang Z, Guo X, Liu J, Sun X. Measuring parents’ acceptance of non-national immunization program vaccines for children and its influencing factors during the COVID-19 pandemic in Shanghai, China. Human Vaccines & Immunotherapeutics. 2022;18(5):2069427. doi:10.1080/21645515.2022.2069427. 35727599 PMC9302520

[28] Ganczak M, Dmytrzyk-Daniłów G, Karakiewicz B, Korzeń M, Szych Z. Determinants influencing self-paid vaccination coverage, in 0–5 years old Polish children. Vaccine. 2013;31(48):5687–5692. doi:10.1016/j.vaccine.2013.09.056. 24120549

