# Supplementary material for: Administration of non-national immunization program vaccines for children under six in a rural county, Henan Province: Did costs matter?
Source: Hum Vaccin Immunother. 2025 Jan 21;21(1):2454744. doi: 10.1080/21645515.2025.2454744 (PMC12934159; doi:10.1080/21645515.2025.2454744)
Supplement: Additional file.docx [file KHVI_A_2454744_SM9244.docx]

**Supplementary Table 1 Caregiver’s knowledge of non-NIP vaccines schedule in a rural county of Henan, 2022**

|  | Total  (n=1051) | | | Left behind  (n=392) | | | | | Non-left-behind  (n=659) | | | | | | | χ2 | | P |
| --- | --- | --- | --- | --- | --- | --- | --- | --- | --- | --- | --- | --- | --- | --- | --- | --- | --- | --- |
|  | N | | % | | | N | | % | | | N | | | % | |  |  |  |
| Multi-dose vaccines can only effectively prevent disease after the last dose. | | | | | | | | | | | |  | | | | 26.844 | | <0.001 |
| Ture | 875 | 83.25 | | | 299 | | 76.28 | | | 576 | | | 87.41 | |  | |  | |
| FALSE | 46 | 4.38 | | | 18 | | 4.59 | | | 28 | | | 4.25 | |  | |  | |
| Don’t know | 130 | 12.37 | | | 75 | | 19.13 | | | 55 | | | 8.35 | |  | |  | |
| Children should be vaccinated strictly according to the schedule. | | | | | | | | | | | |  | | | | 1.854 | | 0.396 |
| Ture | 1039 | 98.86 | | | 389 | | 99.23 | | | 650 | | | 98.63 | |  | |  | |
| FALSE | 9 | 0.86 | | | 3 | | 0.77 | | | 6 | | | 0.91 | |  | |  | |
| Don’t know | 3 | 0.29 | | | 0 | | 0 | | | 3 | | | 0.46 | |  | |  | |
| Vaccination may be unsuitable or delayed based on a child's health. | | | | | | | | | | | |  | | | | 2.088 | | 0.352 |
| Ture | 1033 | 98.29 | | | 388 | | 98.98 | | | 645 | | | 97.88 | |  | |  | |
| FALSE | 11 | 1.05 | | | 3 | | 0.77 | | | 8 | | | 1.21 | |  | |  | |
| Don’t know | 7 | 0.67 | | | 1 | | 0.26 | | | 6 | | | 0.91 | |  | |  | |

**Supplementary Table 2 Caregiver’s information source of non-NIP vaccination-related knowledge in a rural county of Henan, 2022**

|  | Total | | Left-behind | | Non-left-behind | | χ2 | P |
| --- | --- | --- | --- | --- | --- | --- | --- | --- |
|  | (n=1051) | | (n=392) | | (n=659) | |  |  |
|  | N | % | N | % | N | % |  |  |
| Medical staff | 956 | 90.96 | 357 | 91.07 | 599 | 90.9 | 0.009 | 0.923 |
| Mobile applications and internet | 754 | 71.74 | 305 | 77.81 | 449 | 68.13 | 11.343 | 0.001 |
| Local offline health education | 563 | 53.57 | 180 | 45.92 | 383 | 58.12 | 14.708 | <0.001 |
| Relatives, friends, and other parents | 283 | 26.93 | 106 | 27.04 | 177 | 26.86 | 0.004 | 0.949 |
| TV broadcasts and newspapers | 222 | 21.12 | 82 | 20.92 | 140 | 21.24 | 0.016 | 0.900 |
| School | 138 | 13.13 | 46 | 11.73 | 92 | 13.96 | 1.068 | 0.301 |
